# Expression of a Splice Variant of CYP26B1 in Betel Quid-Related Oral Cancer

**DOI:** 10.1155/2014/810561

**Published:** 2014-07-07

**Authors:** Ping-Ho Chen, Ka-Wo Lee, Cheng-Chieh Hsu, Jeff Yi-Fu Chen, Yan-Hsiung Wang, Ker-Kong Chen, Hui-Min David Wang, Hurng-Wern Huang, Bin Huang

**Affiliations:** ^1^School of Dentistry, College of Dental Medicine, Kaohsiung Medical University, No. 100 Shih-Chuan 1st Road, Kaohsiung 80708, Taiwan; ^2^Cancer Center, Kaohsiung Medical University Hospital, No. 100 Shih-Chuan 1st Road, Kaohsiung 80708, Taiwan; ^3^Department of Otolaryngology, Kaohsiung Medical University Hospital, No. 100 Shih-Chuan 1st Road, Kaohsiung 80708, Taiwan; ^4^Department of Otolaryngology, College of Medicine, Kaohsiung Medical University, No. 100 Shih-Chuan 1st Road, Kaohsiung 80708, Taiwan; ^5^Institute of Biomedical Sciences, National Sun Yat-sen University, No. 70 Lienhai Road, Kaohsiung 80424, Taiwan; ^6^Department of Biotechnology, Kaohsiung Medical University, No. 100 Shih-Chuan 1st Road, Kaohsiung 80708, Taiwan; ^7^Orthopaedic Research Center, College of Medicine, Kaohsiung Medical University, No. 100 Shih-Chuan 1st Road, Kaohsiung 80708, Taiwan; ^8^Division of Conservative Dentistry, Department of Dentistry, Kaohsiung Medical University Hospital, No. 100 Shih-Chuan 1st Road, Kaohsiung 80708, Taiwan; ^9^Department of Fragrance and Cosmetic Science, Kaohsiung Medical University, No. 100 Shih-Chuan 1st Road, Kaohsiung 80708, Taiwan; ^10^Department of Biomedical Science and Environmental Biology, College of Life Science, Kaohsiung Medical University, No. 100 Shih-Chuan 1st Road, Kaohsiung 80708, Taiwan; ^11^Department of Biological Sciences, National Sun Yat-sen University, No. 70 Lienhai Road, Kaohsiung 80424, Taiwan

## Abstract

Betel quid (BQ) is a psychostimulant, an addictive substance, and a group 1 carcinogen that exhibits the potential to induce adverse health effects. Approximately, 600 million users chew a variety of BQ. Areca nut (AN) is a necessary ingredient in BQ products. Arecoline is the primary alkaloid in the AN and can be metabolized through the cytochrome P450 (CYP) superfamily by inducing reactive oxygen species (ROS) production. Full-length CYP26B1 is related to the development of oral pharyngeal cancers. We investigated whether a splice variant of CYP26B1 is associated with the occurrence of ROS related oral and pharyngeal cancer. Cytotoxicity assays were used to measure the effects of arecoline on cell viability in a dose-dependent manner. *In vitro* and *in vivo* studies were conducted to evaluate the expression of the CYP26B1 splice variant. The CYP26B1 splice variant exhibited lower expression than did full-length CYP26B1 in the human gingival fibroblast-1 and Ca9-22 cell models. Increased expression of the CYP26B1 splice variant was observed in human oral cancer tissue compared with adjacent normal tissue, and increased expression was observed in patients at a late tumor stage. Our results suggested that the CYP26B1 splice variant is associated with the occurrence of BQ-related oral cancer.

## 1. Introduction

Betel quid (BQ) chewing is an emerging health-associated problem in Asia and among diverse migrant populations in western countries. Approximately, 600 million people worldwide chew BQ [1]. BQ is a psychostimulant and an addictive carcinogenic substance [2, 3]. The areca nut (AN) is the primary ingredient in various BQ products, and the International Agency for Research on Cancer (IARC) has classified the AN as a group I human carcinogen based on findings indicating that it is associated with an elevated risk for oral potentially malignant disorders (OPMDs, such as leukoplakia, oral submucous fibrosis, erythroplakia, and lichen planus) and cancers of the oral cavity, pharynx, and esophagus [3–7].

In Taiwan, the quantity of BQ production increased approximately 44-fold from 1961 to 2001 [3]. In Taiwan alone, 2 million people are habitual BQ users, accounting for 10% of the island's Han Chinese population. BQ is typically used by men (men: 16.5%; women: 2.9%) [8], aboriginal people, blue-collar workers, people with low levels of education, and smokers and drinkers. A recent intercountry ABC study indicated that chewing rates among men (15.6%) were significantly higher than those among women (3.0%) in Taiwan [9]. In 2010, the age-standardized incidence rate of oral and pharyngeal cancer in Taiwanese men, adjusted based on the 2000 world population, was 40.56 per 100 000, ranking as the fourth most prevalent cancer [10]. The age-standardized mortality rate for oral and pharyngeal cancer was 14.71 per 100 000, indicating that this cancer also ranks as the fourth highest cause of cancer deaths.

BQ, the fourth most frequently consumed psychoactive substance worldwide (after caffeine, nicotine, and alcohol), is a masticatory mixture combining the AN, betel leaf, slaked lime, and locally varied flavorings [11]. Our previous results indicated that chewing BQ may generate significantly reactive oxygen species, such as the hydroxyl radical, which may result in oxidative damage in the oral mucosa [12]. In serum-starved oral cells, the AN extract induces pyknotic necrosis by increasing reactive oxygen species (ROS) levels [13]. Arecoline is the most abundant alkaloid in the AN and is known to induce cytotoxicity* in vivo* and* in vitro* in mammalian cells [14–16]. A previous study indicated that arecoline can induce cell cytotoxicity, cycle arrest, and apoptosis in human endothelial cells [17]. In human cells, arecoline was reported to induce the production of ROS [18], which are capable of inducing nucleotide modification and the generation of cellular 8-hydroxy-2′-deoxyguanosine-induced oxidative DNA damage [19]. A previous report indicated that all-trans retinoic acid (at-RA) induces NADPH oxidase-mediated ROS generation in granulocyte-differentiated HL60 cells [20]. In rat Sertoli cells, at-RA can generate apoptosis [21] by inducing ROS production [21, 22].

Previous studies have indicated that CYP26B1, a member of the cytochrome P450 (CYP) superfamily, is a primary at-RA metabolizing enzyme. The at-RA is a structure of retinoic acid (RA), which is an active derivative of vitamin A. Dietary retinol (vitamin A) has been metabolized into at-RA. Numerous reports have suggested that RA deficiency may be associated with carcinogenesis [23–26]. Insufficient retinol intake is related to hyperkeratosis and hyperplasia of the oral mucosa [25]. A report indicated that remission in OPMD patients with BQ chewing habits treated with RA may result from suppression of the promoting action of AN ingredients rather than inhibition of tumor initiation [27].

Our previous studies indicating that arecoline induces both* in vivo* and* in vitro* suggest that the CYP26B1 variants play a vital role in BQ-related oral and pharyngeal cancers [7, 28]. We investigated whether susceptible CYP26B1 genes and, particularly, their splicing variants are associated with oral and pharyngeal cancer.

## 2. Methods

### 2.1. Cytotoxicity Assay

Detailed information on the cell culturing procedure is provided previously [29, 30]. We obtained normal human gingival fibroblasts (HGF-1) from the American Type Culture Company (ATCC number CRL-2014) and Ca9-22 cells (a cell line of oral epidermal gingival squamous carcinoma) from the Japanese Collection of Research Bioresources Cell Bank (JCRB number JCRB0625). The arecoline-conditioned medium (0.05–0.8 mM) was freshly prepared from arecoline hydrobromide (Sigma) in a growth medium (DMEM/F12). Cells were seeded into 96-well plates at a density of 10^4^ cells per well for 1 day and were then treated with various concentrations (0, 0.05, 0.1, 0.2, 0.4, and 0.8 mM) of arecoline for 24 h in a CO_2_ incubator. MTT (Sigma) solution (5 mg/mL) was added to the cells and incubated for 2 h at 37°C. After the culture medium was removed, the cells were dissolved using DMSO, and absorbance was detected at 570 nm in an ELISA reader (Bio Tek el800), with a reference wavelength of 630 nm. The data are presented as the percentage of viable cells compared with the controls.

### 2.2. Western Blotting Analysis

Details on the method used for western blotting are provided in our previous report [7]. Mouse antihuman CYP26B1 monoclonal antibodies (Abnova, Taipei City, Taiwan) were used as the first antibody at a 1 : 1000 dilution, and a horseradish-peroxidase conjugated antimouse antibody (Cell signaling Co.) was used as the second antibody at a 1 : 1000 dilution. Both antibodies were dissolved in 3% nonfat dry milk (GE Healthcare, Uppsala, Sweden) in 0.05% TBST (8 g NaCl, 0.2 g KCl, 3 g Tris, 0.5% Tween 20, dd H_2_O to 1 L, and pH 7.4). Peroxidase activity was detected using an ECL detection kit (GE Healthcare) and recorded using the HyperFilm TMMP (GE Healthcare).

### 2.3. Study Participants

The Department of Otorhinolaryngology and the Dentistry Department of Kaohsiung Medical University (KMU) Hospital recruited 10 male oral cancer patients. This study was approved by the institutional review board (IRB) of the Human Experiment and Ethics Committee of KMU (KMU-IRB-950070 and KMU-IRB-950072). The volunteers agreed to provide written informed consent, provide oral cancerous tissue (necessary for resection), and complete questionnaires. Pairs of cancer tissue and adjacent noncancerous oral tissue were collected from patients with oral cancer for use in a CYP26B1 gene expression assay. Pathologists or surgeons histologically confirmed that all cases were cases of oral cancer. Demographic data, information regarding previous substance use, and clinical information on the participants were collected by administering a questionnaire and analyzed.

### 2.4. Identification of CYP26B1 and CYP26B1 Splice Variant mRNA Expression in HGF-1 and Ca9-22 Cells

The mRNA levels of CYP26B1 and expression levels of the CYP26B1 splice variant in HGF-1 and Ca9-22 cells treated with arecoline were determined using semiquantitative PCR. Briefly, total RNA was isolated using a Trizol kit (Roche). The integrity and quality of total RNA were determined using DU 800 (Beckman Coulter). Total RNA was subjected to High-Capacity cDNA Reverse Transcription Kits (Applied Biosystems) to obtain complementary DNA (cDNA). PCRs were performed to investigate the expression of the genes. We designed the PCR primers in CYP26B1 gene exon 1 and exon 3 positions with oligonucleotide primers directed against cDNA sequences of CYP26B1 (forward 5′-TCTTTGAGGGCTTGGATCTG-3′, reverse 5′-GGATCACCAGCTGGATCTTG-3′). The PCRs were performed as follows: initial denaturation at 94°C for 5 min; 35 cycles of denaturation at 94°C for 30 s; annealing at 53°C for 30 s; extension temp. 72°C for 1 min; and holding at 72°C for 7 min to complete the reaction. The RT-PCR products were electrophorized in 2% agarose gel and then stained with ethidium bromide. The semiquantitation of mRNA expression was determined by subjecting the RT-PCR products to densitometry (Epson 1640 U). The cDNA (about 0.2 *μ*g) was used as a template in a PCR amplification reaction to obtain gene products of 2 lengths: full-length CYP26B1 composed of 464 nucleotides and a CYP26B1 splice variant composed of 244 nucleotides.

The results of sequencing the 2 gene products by using NCBI BLAST indicated that the products were 99% to 100% similar (Figures 2(a) and 2(b)). The excitation intensity of the band from the agarose gel revealed that, even when the same cDNA concentration and number of cDNA reaction cycles were applied, CYP26B1 gene expression in the normal HGF-1 cell line was much lower than that of oral cancerous Ca9-22 cells.

### 2.5. Statistical Analysis

The one-way ANOVA and Bonferroni multiple comparison test were applied to analyze the relative fold change in the treatment groups compared with the control (HGF-1 or Ca9-22 without arecoline treatment), and a statistically significant difference (*P* < 0.05) is indicated using an asterisk. All statistical analyses were performed using the IBM SPSS Statistics 19, and the results were considered statistically significant when *P* < 0.05.

## 3. Results

### 3.1. Cell Viability of HGF-1 and Ca9-22 Cells

**Figure 1:**
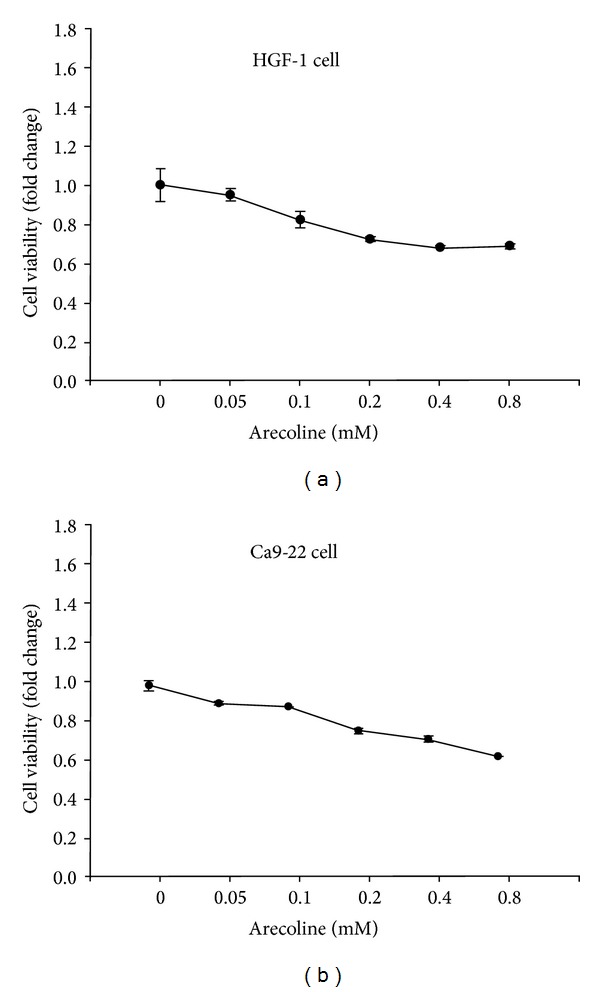
Effect of arecoline on the viability of HGF-1 and Ca9-22 cells. Different concentrations of arecoline were incubated with HGF and Ca9-22 cells for 24 h. Cell viability was determined using an MTT assay. (a) Arecoline induced cell death in various concentrations of arecoline in HGFs. (b) Arecoline induced cell death in various concentrations of arecoline in Ca9-22 cells.

**Figure 2:**
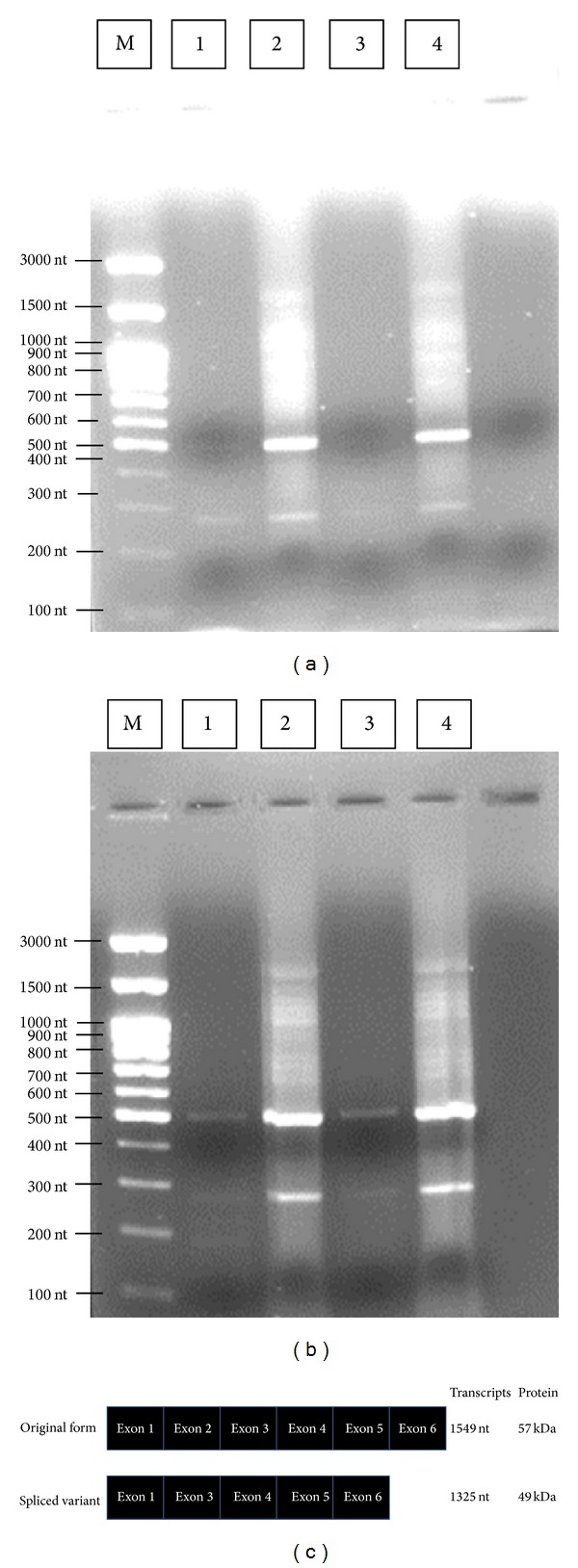
Expression of full-length CYP26B1 (57 kDa) and the CYP26B1 splice variant (49 kDa) in HGF-1 and Ca9-22 cell lines. CYP26B1 and the CYP26B1 splice variant were examined using RT-PCR. (a) Expression of the CYP26B1 gene in nontreated HGF-1 cells. (b) Expression of the CYP26B1 gene in nontreated Ca9-22 cells. (c) Full-length CYP26B1 and the splice variant. M: marker; lane 1: template 2 *μ*g; lane 2: template 20 *μ*g; lane 3: template 2 *μ*g; lane 4: template 20 *μ*g.

We used the MTT assay to evaluate cell viability following exposure to 6 concentrations (0, 0.05, 0.1, 0.2, 0.4, and 0.8 mM) of arecoline for 24 h. Cell survival gradually decreased after increasing the arecoline concentration in a dose-dependent manner.  Figure 1(a) shows that arecoline reduced cell survival predominantly in a dose-dependent manner, and cell survival approached approximately 70% at arecoline concentrations of 0.4 mM and 0.8 mM. When the HGF-1 cells were treated with 0.05, 0.1, 0.2, 0.4, and 0.8 mM arecoline for 24 h, cell survival was 94%, 82%, 72%, 68%, and 68% compared with the control group, which did not receive arecoline treatment. This result was also observed in cultured Ca9-22 cells, of which viability remarkably decreased and reached about 60% at the concentration of 0.8 mM (Figure 1(b)). When the Ca9-22 cells were treated with 0.05, 0.1, 0.2, 0.4, and 0.8 mM arecoline for 24 h, cell survival was 90%, 89%, 76%, 72%, and 63% compared with the control group.

### 3.2. Expression of the Full-Length (57 kDa) CYP26B1 and CYP26B1 Splice Variant (49 kDa) in Cells

**Figure 3:**
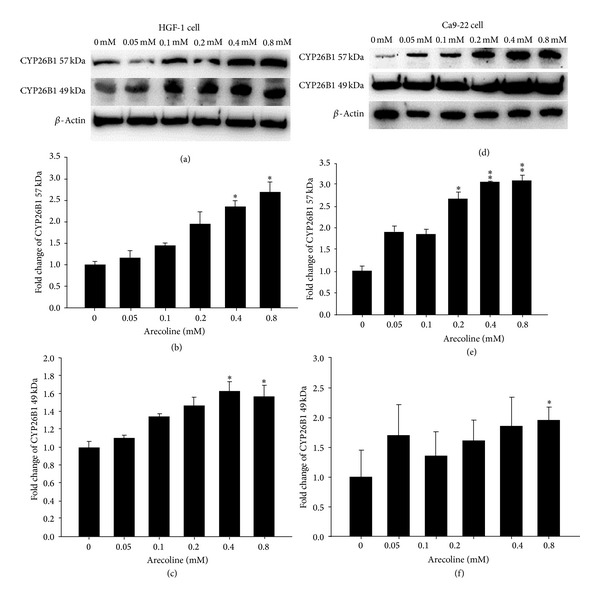
Expression of CYP26B1 induced by arecoline for 24 h in cultured HGF-1 and Ca9-22 cells. (a) Expression of full-length CYP26B1 (57 kDa) and the splice variant (49 kDa) in HGF-1 cells exposed to arecoline at various concentrations compared with the control, which did not receive arecoline treatment; (b) densitometric analysis of the expression of full-length CYP26B1 bands in HGF cells; (c) densitometric analysis of the expression of the CYP26B1 splice variant in HGF-1 cells; (d) expression of full-length CYP26B1 (57 kDa) and the splice variant (49 kDa) in Ca9-22 cells exposed to arecoline at various concentrations compared with the control; (e) densitometric analysis of the expression of full-length CYP26B1 bands in Ca9-22 cells; (f) densitometric analysis of the expression of the CYP26B1 splice variant in Ca9-22 cells. Mean ± SD; *P < 0.05; **P < 0.01; *n* = 3.

In normal HGF-1 cultures treated with various concentrations of arecoline, the expression of the full-length CYP26B1 protein and the splice variant exhibited significant upregulation compared with the control according to western blot analysis (Figure 3(a)). In normal HGF-1 cultures treated with doses of 0.4 and 0.8 mM arecoline, the expression of the full-length CYP26B1 protein exhibited significant upregulation (2.4-fold and 2.7-fold, separately) compared with the control (Figure 3(b)). Expression of the CYP26B1 splice variant exhibited significant minor upregulation (1.6-fold and 1.5-fold, separately) at doses of 400 and 800 *μ*M arecoline in normal HGF cultures compared with the control (Figure 3(c)).

Similarly, in cancerous oral Ca9-22 cell cultures, the expression of full-length CYP26B1 and the splice variant exhibited significant upregulation compared with the control (Figure 3(d)). In cancerous oral Ca9-22 cell cultures treated with arecoline (0.2 mM, 0.4 mM, and 0.8 mM), we observed significantly increased expression of full-length CYP26B1 (the fold changes were 2.7-fold, 3.1-fold, and 3.1-fold, resp.; Figure 3(e)). Expression of the CYP26B1 splice variant significantly increased only in cancerous oral Ca9-22 cell cultures treated with 800 *μ*M arecoline (Figure 3(f)).

### 3.3. Expression of the CYP26B1 Splice Variant (49 kDa) in the Study Population

**Figure 4:**
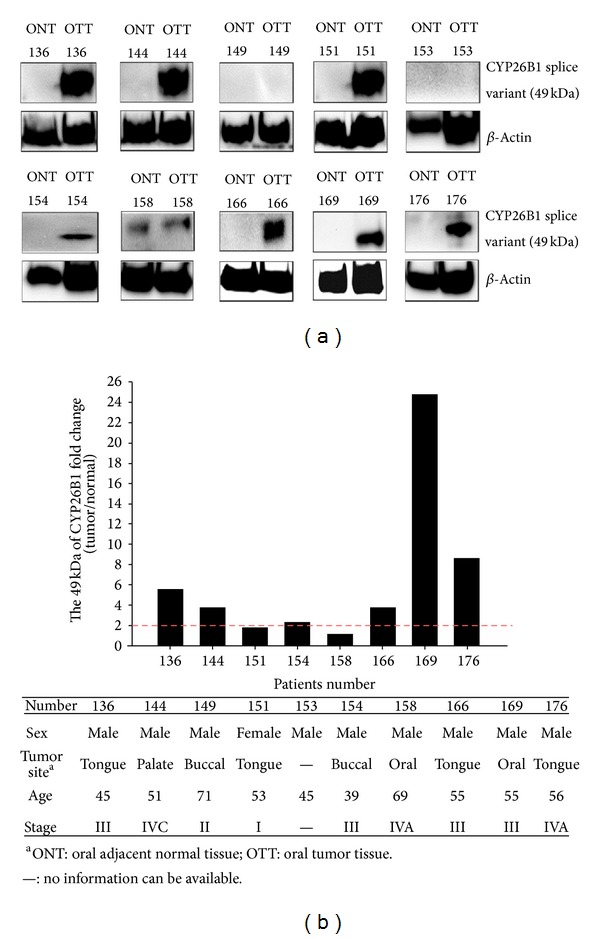
Protein level of the CYP26B1 splice variant (49 kDa) in human OSCC tissue (T) and adjacent normal tissue (N). (a) Level of CYP26B1 splice variant protein expression in 10 pairs of oral tumor tissue and adjacent tissue; *β*-actin was used as a control for protein loading and detection. (b) Expression protein fold change level of CYP26B1 in 10 pairs of oral tumor tissue compared with the adjacent normal tissue.

The oral cancer tissue exhibited consistent upregulation in the protein levels of the CYP26B1 splice variant.  Figure 4(a) shows that the protein expression of the CYP26B1 splice variant (*N* = 8 paired sample) was an average of 6.44-fold higher in human oral squamous cell carcinoma (OSCC) tissue than in the adjacent noncancerous tissue. When a 2.0-fold change was defined as the threshold, 6 of 8 (75%) paired samples were considered significantly upregulated (>2-fold) in a range from 2.32-fold to 24.72-fold. The cases that did not exhibit significant upregulation were cases 151 (1.74-fold) and 158 (1.11-fold). The clinical characteristics of the patients are shown in Figure 4(b). The results indicated that patients with late-stage (stage III or stage IV) carcinoma exhibited a greater than 2-fold change in expression of the CYP26B1 splice variant.

## 4. Discussion 

BQ chewing is a popular habit among Taiwanese men. Although BQ ingredients vary among regions worldwide, the AN is the primary component. Our previous study indicated that variants of full-length CYP26B1 may be associated with the occurrence of BQ-related OSCC [7]. In addition, a previous study indicated that RA metabolizing enzymes CYP26B1 are overexpressed significantly in colorectal cancer and that CYP26B1 is significantly associated with the prognosis of colorectal cancer patients [31]. According to our thorough review of relevant research, no previous study has explored the relationship between the expression activity of the CYP26B1 splice variant and oral cancer.

Arecoline is a primary alkaloid in the AN. The IARC stated that evidence obtained from animal experiments strongly indicates that the AN is carcinogenic and that limited evidence indicating that arecoline is carcinogenic has been obtained from animal experiments [3]. In this experiment, normal oral cells (HGF-1) and oral cancer cells (Ca9-22) were used as cell models to explore whether arecoline treatment causes cell toxicity and affects cell survival. At 0.8 mM arecoline, the cell viability of HGF-1 cells was 68% and Ca9-22 cell survival was 63% compared with the control group. Using a cell viability curve, we observed that increasing the concentration of arecoline resulted in declining cell survival. Previous studies have shown that arecoline can cause carcinogenicity, cytotoxicity, immunotoxicity, and genotoxicity [32–34]. Stimulated human peripheral blood mononuclear cells and human keratinocytes can secrete and release cytokines, causing arecoline to regulate inflammatory processes [35, 36]. A previous report indicated that arecoline induced ROS generation, suggesting that oxidative stress plays a role in arecoline-mediated cell death, gene regulation, and inflammatory procedures in human keratinocytes [34].

CYP systems are considered to contribute to detoxification of arecoline and the AN during phase I metabolism [37]. A metabolic map of arecoline in mice revealed that CYP or a flavin-containing monooxygenase might create 3 forms of* N*-oxide metabolites or arecoline [38]. Although the CYP26B1 splice variant lacks exon 2 in the coding region, it still has the ability to downgrade at-RA [39]. A previous study indicated that full-length CYP26B1 plays a crucial role in the catabolism of at-RA and regulation signaling [40], whereas the splice variant of CYP26B1 exerts slight or no influence on at-RA regulation [39].

The expression levels of CYP26B1 and its splice variant were examined according to both mRNA and protein levels in HGF-1 and Ca9-22 cells, which represented normal and cancerous oral cells. We observed that arecoline can induce expression of both forms of CYP26B1 and that this induction rate is greater for full-length CYP26B1 in cancer cells (i.e., Ca9-22). The expression of full-length CYP26B1 was higher than that of the splice variant of CYP26B1 in HGF-1 and Ca9-22 cells treated with arecoline. Our arecoline cell toxicity data obtained from the MTT assay indicated that, when the quantity of CYP26B1 is higher, Ca9-22 cells are more susceptible to arecoline. This* in vitro* observation revealed that the expression of full-length CYP26B1 plays a greater role than does that of the CYP26B1 splice variant and is consistent with a previous report [39]. Using paired human oral cancer tissues with long-term chewing habits, we confirmed that the expression of the CYP26B1 splice variant was consistently higher in oral cancerous tissues than in adjacent noncancerous tissues. Expression of the CYP26B1 splice variant was 2-fold higher in 6 of 8 cancer tissues (75%) than in adjacent noncancerous tissues. Our results indicated that the protein expression of cases 136, 144, 154, 166, 169, and 176 was more than 2-fold (the greatest fold change was 24.7), and their stages were late (stage III or stage IV).

Arecoline, which is known to exert adverse effects, is correlated with BQ exposure [41] and can produce cytotoxicity, possibly causing the monooxygenase enzyme system (such as CYP26B1) to continue the oxidative metabolism of arecoline. The 6-ring structure of at-RA, which is metabolized by CYP26B1, is similar to that of arecoline. The mechanism through which CYP26B1 metabolizes arecoline remains unknown. We speculate that CYP26B1 plays a role in arecoline catalysis during phase I metabolism. Arecoline can stimulate the high expression of CYP26B1 or cellular at-RA, causing cancer cells to carry on the apoptosis pathway and to maintain balance and survival, resulting in CYP26B1 overexpression. However, the physiologic importance of the increased induction rate and quantity of CYP26B1 by arecoline, accompanied by ROS generation in oral cancer cells, requires further investigation.

A limitation of this study was the relatively small sample size. Because this research was more difficult to get paired tissue specimen, we suggested that the future research needs a larger number of samples to confirm expression of a splice variant of CYP26B1 in betel quid-related oral cancer.

## 5. Conclusion

We observed an association between a splice variant of CYP26B1 and BQ-related oral cancer, thus providing insight into the molecular mechanism of full-length CYP26B1 and a splice variant of CYP26B1 as well as their joint effects in the development of BQ-related oral cancer. This research may provide a valuable contribution into the importance of screening tests for the chemoprevention strategies and effective treatments for BQ-related oral cancers.
